# Enhanced humidity sensing performance of LiF-doped MgTiO_3_ ceramics via spark plasma sintering

**DOI:** 10.1016/j.heliyon.2024.e33999

**Published:** 2024-07-02

**Authors:** Ahmad Kassas, Israa Zahwa, Bassam Hussein, Jérôme Bernard, Céline Lelièvre, Mohamed Mouyane, Jacques Noudem, David Houivet

**Affiliations:** a The International University of Beirut, School of Engineering, Department of Industrial Engineering, Beirut, Lebanon; b LUSAC-EA4253, Université de Caen Basse-Normandie, Rue Louis Aragon, BP 78, 50130, CHERBOURG-OCTEVILLE, France; c Lebanese International University, School of Engineering, Department of Industrial Engineering, Bekaa, Lebanon

**Keywords:** Electroceramics, Spark plasma sintering, Dielectric materials/properties, Sensors

## Abstract

The fabrication of low-porosity ceramics for humidity sensing is crucial to prevent moisture entrapment, which poses significant challenges. Using Spark Plasma Sintering (SPS), we successfully densified fine-grained ceramic materials based on LiF-doped MgTiO_3_, comparing them with conventionally sintered counterparts. Structural and microstructural analyses, employing X-ray diffraction and Scanning Electron Microscopy (SEM), were conducted. Investigation into electrical and dielectric properties' variations concerning humidity levels was conducted and revealed that SPS sintered ceramics exhibit heightened sensitivity to moisture, as evidenced by resistivity, capacitance, and response time measurements. The implications of these results are discussed in depth, highlighting the potential of SPS as a promising method for fabricating humidity sensors with improved performance and reduced porosity-related issues.

## Highlights

• Sintering temperature of MgTiO_3_ + 2 wt% LiF (ML2) lowered to 800 °C by SPS.
• The SPS ceramic has higher density and lower porosity compared to the conventional sintering.
• The ML2 SPS ceramic shows high sensitivity, Surface detection of moisture with total fast desorption.
• It can be used as capacitive humidity switch and/or as resistive humidity sensor.

## 1 Introduction

The improvement and innovation of sensing materials is a challenge nowadays due to their important role in different fields, such as environmental monitoring, agriculture, food processing, medicine, meteorology and beyond. Recently, various materials, including polymer [1], electrolytic [2], and ceramic materials [3], have been explored for making the sensitive components of humidity sensors [4,5]. Ceramic based humidity sensors are particularly valued for their heightened stability across different chemical environments, wide operational temperature range, and rapid response to humidity fluctuations [6]. Numerous ceramic materials like ZrP [7], CoCr_2_O_4_ [8], Co_1-x_Cd_x_Cr_2_O_4_ [9], CuO [10], Hf_1-x_(In_0.5_Nb0.5)_x_O_2_ [11], CaCu_3_Ti_4_O_12_ [12], Li_2.10_Al_1.23_Si_1.54_Cl_0.05_O_5.95_ [13], MgCr_2_O_4_–TiO_2_ [14], ZnCr_2_O_4_–ZnO [15], La–Ti–V–O glass ceramics [16], LaFeO_3_ [17], La_0.7_Sr_0.3_MnO_3_ [18] have been investigated in this regard. Generally, ceramic sensors offer cost-effective solutions, where the composition and the microstructure are readily controlled [19].

The impedance change of the ceramic sensors at different humidity ratio is caused through the adsorption-desorption of water molecules on oxide surfaces; a phenomenon tied to the morphology of the sensing element [17,20,21]. The water vapor condensation behavior, result of capillary action, is influenced by ceramic pore size and distribution [[21], [22], [23]]. Thus, porosity exerts a dual effect on humidity sensitivity; while increasing porosity enhances specific surface area, facilitating more active sites for water molecule adsorption, it also exacerbates adsorption-desorption hysteresis by trapping water molecules within pores [24].

Porosity levels in ceramics are modulated during densification or sintering processes which involves the transition of compact individual solid particles into a coherent ceramic structure governed by various diffusion phenomena. Sintering methods can be categorized as conventional or non-conventional including Field Assisted Sintering (FAST), Field Assisted Rapid Sintering (FARS), Hot Pressure (HP) sintering, Hot Iso-Pressure (HIP) sintering, and Cold sintering Process [[25], [26], [27], [28], [29]]. Techniques like Spark Plasma Sintering (SPS) offer significant advantages enabling rapid and efficient densification at lower temperatures and producing controlled microstructures leading to materials with improved properties and extended areas of application [30] compared to conventional methods [[31], [32], [33]].

SPS facilitates high densification and fine-grained ceramics through [[33], [34], [35]].

i. High heating rates; minimizing the duration spent at lower temperatures where diffusion dominates.
ii. Applied pressure; intensifying defect gradients within powder particles and enhancing sintering energy.

Conventional sintering of MgTiO_3_ with LiF additive has been explored and showed how LiF impacted moisture sensitivity, induced structural, and microstructural change [[36], [37]]. Specifically, MgTiO_3_ doped with 2 wt% of LiF exhibited promising sensitivity to moisture rendering it suitable for humidity sensing applications and switches. However, excessive porosity and uncontrolled distribution adversely affected electrical and dielectric responses to moisture [37].

This study focuses on the preparation of high-density, fine-grained ceramic materials based on MgTiO_3_ doped with 2 wt% of LiF through Spark Plasma Sintering, comparing them to conventionally sintered counterparts. The structural development, microstructure, and humidity sensing properties of the prepared ceramics are discussed.

## 2 Material and methods

MgTiO_3_ powder, Mg/Ti = 1.025, was synthesized via the conventional solid-state route. Initially, MgO (Cerac 99 %) and TiO_2_ (anatase Prolabo 99 %) powders were mixed for 1 h in demineralized water by attrition milling (Dyno Mill KDLA, BACHOFEN, Switzerland) with yttrium stabilized zircon balls (0.8 mm diameter, YTZ grinding media TOSOH). Subsequently, Powders were calcined in air at 1000 °C for 1 h to obtain the MgTiO_3_ ilmenite phase which was then confirmed by X-ray analysis.

A 2 wt% (equivalent to 9 % molar ratio) of lithium fluoride was introduced to the MgTiO_3_ powder followed by milling in a planetary agitator (Pulverisette FRITCH) and resulting in the formation of ML2 mixture. The ML2 SPS sintering was carried out with the "Spark Plasma Sintering (SPS)" device (Model: HP D 25/1, Fine Ceramic Technologies (FCT)).

For conventional sintering, ML2 powder was pressed at 2000 kg cm^−2^ (at a pressure of 74 MPa) to form pellets 25 mm diameter and around 2 mm thick. The pellets were sintered at 900 °C in a tubular oven under oxygen flow with 150 °C h^−1^ heating and cooling rates and 1 h dwell time.

X-ray diffraction patterns were collected using a SIEMENS D 5005 diffractometer (CuKα, *λ* = 1.5405 Å), over a range of 10**°-**80**°** (2*θ*). Interconnected porosity was measured using MICROMERITICS AutoPore III mercury porosimeters. Microstructural analysis of the sintered samples was investigated via scanning electron microscopy (SEM) (HITACHI S-2460-N.

Dielectric and electrical properties were characterized by measuring capacitance and insulation resistance as a function of relative humidity using a hot-cold oven with controlled humidity and temperature system (SECASI technologies SLH 100). Measurements under AC field utilized a multiplexer (Agilent Data Acquisition/Switch Unit 34970A), while an RLC bridge (AUTOMATIC PROGRAMMABLE FLUKE PM6306 RCL Meter) measured capacitance and resistance changes with a current regulator adjustable from 60 Hz to 1 MHz. DC electric field resistance measurements were conducted using a Megohmeter (Sefelec M1500P) with a voltage gradient of 70 V mm^−1^ for 10 min to stabilize the values.

The response time of a humidity sensor is the time required for a sensor output to change from a current state to a final stable value within a tolerance band of the correct final value.

To assess response time under AC field, a custom device was developed. This device comprised two compartments, each equipped with humidity and temperature sensors. One compartment maintained a dry atmosphere (4 % ± 2.5 % RH), while the other maintained a saturated environment (95 % ± 2.5 % RH). A sample holder with wire connections to the RLC bridge facilitated continuous capacitance measurements while a rotating cap allowed the sample transition between the two compartments enabling real-time capacitance variation measurements as a function of time.

## 3 Results and discussion

### 3.1 Structural and microstructure analysis

Investigations into shrinkage and shrinkage rate curves variation as function of the sintering temperature in SPS are crucial for determining reduced sintering temperatures and improved sample densification compared to conventional methods. The ML2 powder was placed in 20 mm diameter graphite mold surrounded by graphite sheets to prevent welding. Prior to heating, the sample was uniaxially pressed at 5 kN in the SPS machine. After that, heat treatment under vacuum atmosphere was carried out at 1200 °C (Temperature was measured using an optical pyrometer) with a dwell time of 10 min and a ramp of 100 °C.min^−1^, while a 50 MPa pressure was applied during the heating.

On the other hand, conventional dilatometric analysis was established and the shrinkage behavior was analyzed up to 1300 °C in air using a TMA92 SETARAM dilatometer with 300 °C.h^−1^ heating and 1200 °C.h^−1^ cooling rates.

**Fig. 1:**
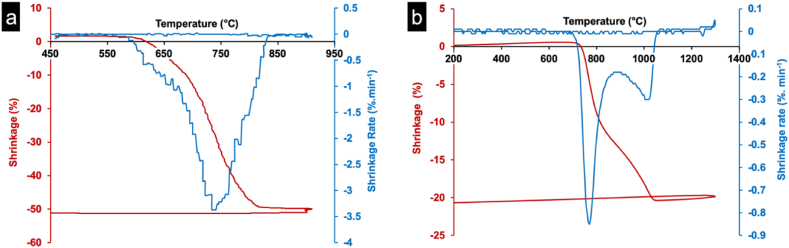
Shrinkage of ML2 powder during spark plasma sintering (a) and conventional sintering (b).

Fig. 1-a illustrates that SPS achieves approximately 52 % shrinkage contrasting with conventional dilatometry's 20 % (Fig. 1-b) thus indicating significantly enhanced densification despite initial high porosity.

Notably, SPS-induced shrinkage occurs predominantly in one direction. The process initiates at 600 °C with a rate of about 1 %.min^−1^ followed by sintering activation at around 734 °C with high shrinkage rate of 3.37 %.min^−1^ attributed to LiF flux agent melting. The shrinkage reaches 50 % at around 832 °C and remains constant.

The shrinkage behaviors of ML2 ceramic prepared by SPS and by conventional sintering show that the onset of sintering by SPS is earlier than conventional sintering. It starts around 600 °C (SPS outer mold temperature) compared to 730 °C in the case of conventional dilatometry. it must be noted that the temperature measured with the pyrometer at the exterior party of the SPS mold is lower than that of the grains. Given the method of measuring the temperature used, the difference can be estimated at 50 °C [[38], [39]]. Additionally, the use of a graphite mold, sensitive to oxidation, precludes oxygen flow, potentially activating sintering via anion vacancy creation. Consequently, SPS achieves densification at lower temperatures prompting adoption of 800 °C for subsequent sintering temperature using a heating rate of 100 °C.min^−1^ under vacuum while applying 50 MPa pressure.

**Fig. 2:**
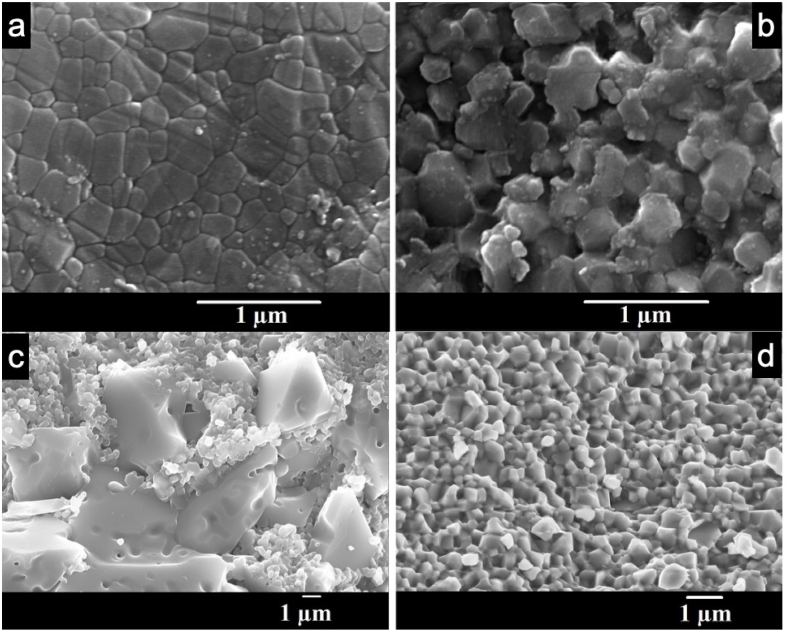
SEM micrographs of the ML2 SPS pellet surface and the fractured pellet before re-oxidation (respectively a and b), and after re-oxidation (respectively c and d).

The microstructure on the surface of the ceramic pellets sintered at 800 °C for 10min prior to oxygen annealing is shown in Fig. 2-a revealing a grain size ranging from 200 to 600 nm similar to the starting powder's average grain size of 240 nm. Cross-sectional analysis (Fig. 2-b) indicates uniform grain sizes throughout the pellet. Notably, grains with identical forms and 120° triple joint grain boundaries are observed and attributed to the rapid sintering process of Spark Plasma Sintering (SPS) within 30 min preventing grain coalescence. Dark coloring in the micrographs is attributed to residual graphite diffusion in the pellets which has been eliminated by annealing the ML2 SPS pellets at 700 °C for 2 h under O_2_ flow in a tubular furnace. This last treatment has another important role as it will fill the anion vacancies in the samples created during the SPS process.

Two distinct grain sizes emerge: surface grains reaching up to 6 μm (Fig. 2-c) and smaller submicron grains. However, small size grains are detected through the pellet volume (Fig. 2-d). Large surface grains may correspond to the formation of the FCC Li_2_MgTiO_4_ phase generated on the surface of pellets prepared by conventional sintering [36], although x-ray analysis fails to detect this due to their low proportion.

Microstructure analysis confirms the sample's dense nature with a measured skeletal density of 3.92 ± 0.01 g cm^−3^ which is consistent with open porosity values (2 % ± 1) from mercury intrusion porosimetry. In contrast, conventionally sintered pellets at 900 °C exhibit a lower skeletal density of 3.86 ± 0.01 g cm^−3^ and higher open porosity (17 % ± 2) highlighting the efficiency of Spark Plasma Sintering in achieving densification and reducing porosity.

**Fig. 3:**
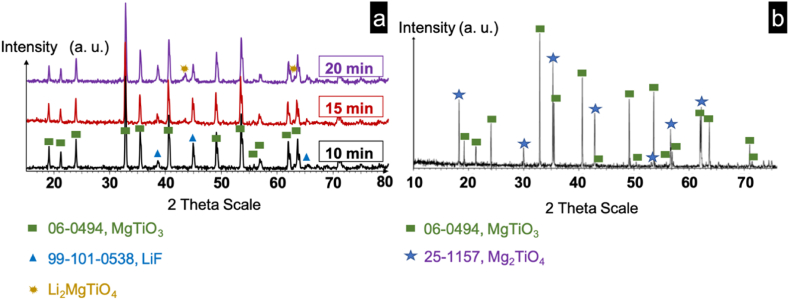
X-ray patterns for ML2 pellets fabricated via different methods: (a) SPS with different dwell times and (b) conventional sintering at 900 °C for 1 h.

After re-oxidation, the surface of SPS-sintered ML2 reveals the presence of the MgTiO_3_ rhombohedral phase and the LiF Face-Centered Cubic phase (FCC). Interestingly, the Mg_2_TiO_4_ and Li_2_MgTiO_4_ phases, typically generated during conventional sintering (Fig. 3-b), are absent on the surface of samples annealed at 800 °C for 10 min (Fig. 3-a). To clarify the crystalline structure of the large grains detected by SEM micrographs (Fig. 2-c) further, we extended the dwell time in the SPS thermal cycle anticipating an increase in phase formation. X-ray diffractograms of samples sintered at 800 °C for varying dwell times (10, 15, and 20 min) are depicted in Fig. 3-a. Increasing the dwell time results in a reduction of FCC LiF Phase intensity, while intensifying the presence of Li_2_MgTiO_4_, is particularly notable at 20 min. These observations echo structural changes observed in conventionally sintered pellets where LiF redistributes throughout the sample during sintering culminating in Li_2_MgTiO_4_ phase formation on the surface [36]. This suggests a similar migration of LiF flux in SPS, facilitating the surface formation of the Li_2_MgTiO_4_ phase.

### 3.2 Electrical and dielectric properties

The electrical and dielectric measurements of the ML2 pellet, featuring an extremely low open porosity percentage prepared via SPS at 800 °C for 10 min were conducted and juxtaposed with those of the ML2 pellet prepared through conventional sintering at 900 °C for 1h.

The sintered pellets surfaces were polished and a gold ink electrodes of 16 mm length and 1 mm width with 0.5 mm spacing was screen printed and annealed in tubular oven under air flow at 800 °C for 15 min with heating and cooling rates of 400 °C.h^−1^.

**Fig. 4:**
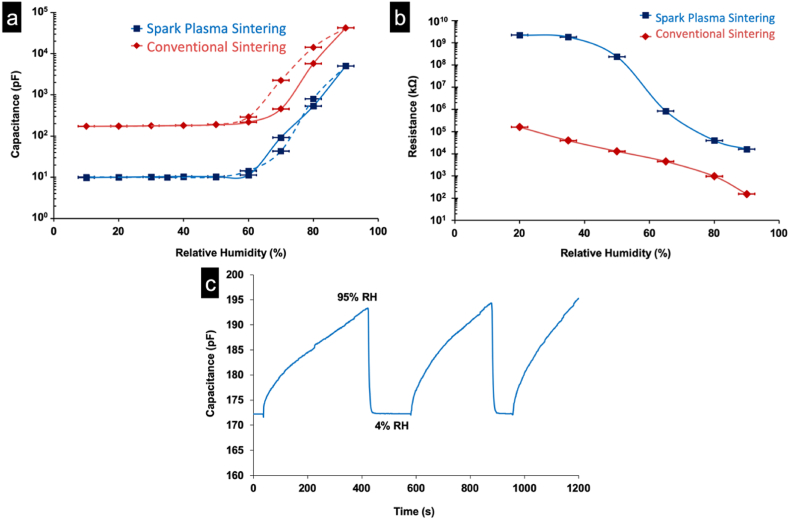
Variations of capacitance (full line adsorption, dotted line desorption) and Resistance against RH % (respectively a and b) Response and recovery plot between 5 % and 95 % RH (c) for the sensing ML2 pellets.

Fig. 4-a depicts the relative capacitance plot, measured at 20 °C, illustrating the relationship between RH ratios for SPS and conventionally sintered ML2 pellets. The measurements were conducted at a frequency of 100Hz. Notably, the capacitance curve for the SPS sample consistently falls below that of the conventionally sintered pellet. This discrepancy arises due to the reduced active surface area of the SPS sample compared to the conventionally sintered one achieved by decreasing open porosity. Consequently, the SPS sample exhibits lower moisture adsorption compared to the conventional case leading to proportionally lower capacitance values.

The SPS sample exhibits a capacitance range increasing from 10 pF at 10 % ± 2.5 RH to 5000 pF at 90 % ± 2.5 RH while the conventional sample shows a change from 175 pF to 40000 pF within the same RH range. Both samples display low sensitivity below 60 % RH. Humidity hysteresis characteristics were analyzed for sensors using SPS ML2 pellets (square symbol) and conventional ML2 pellets (lozenge symbol) at 100Hz as depicted in Fig. 4-a. Full lines represent the adsorption process from low to high relative humidity while dotted lines denote desorption. Hysteresis is minimal during absorption-desorption cycles of the ML2 SPS pellet contrasting with clear hysteresis in conventionally sintered pellets. This discrepancy likely relates to the low porosity of SPS ceramics affecting only the outer surface in capacitance measurements.

However, the resistance values of the SPS pellet (annealed at 20 °C with 35v DC and 10 min measurement time) decrease from 2.10^9^ kΩ to 2.10^4^ kΩ as the relative humidity changes from 20 % to 90 % RH (Fig. 4-b). Conversely, the resistance of the ML2 pellet, produced through conventional sintering, exhibits a range from 2.10^5^ kΩ to 30 kΩ over the same humidity span (20 %–90 % RH) as illustrated in Fig. 4-b. Notably, the resistance of the SPS densified sample surpasses that of the porous sample prepared via conventional sintering owing to higher moisture adsorption across all relative humidity percentages. The resistance curve depicted in Fig. 4-b illustrates that the SPS pellet exhibits lower sensitivity compared to conventional pellets when the humidity ratio is below 30 % RH. This dissimilarity arises from the adsorption of water molecules within two-dimensional networks of cations and anions on the pellet surfaces. At lower relative humidity percentages, a dissociating mechanism of water molecules results in the formation of a non-continuous layer of hydroxyl groups. As the humidity ratio increases, this chemisorbed layer becomes more continuous, activating conduction after 30 % RH. Beyond 55 % RH, a physisorbed layer of water molecules begins to form, causing a rapid decrease in resistance [40]. Conduction at low humidity ratios occurs through the proton jump (H^+^) from one hydroxyl group to another in the chemisorbed layer. Conversely, at higher humidity ratios, conduction is facilitated by the transfer of hydronium ions (H_3_O^+^). The activation energy of H^+^ is greater than that of H_3_O^+^, resulting in higher resistance values at low humidity ratios, which decrease as humidity increases [41]. At approximately 90 % RH, capillary condensation of water molecules forms a liquid-like layer, leading to electrolytic conduction [21], and resistance variation tends towards a limit, becoming sluggish.

Response and recovery characteristics of the SPS pellet in capacitive measurements at a frequency of 100 Hz illustrated in Fig. 4-c. The response time, measured by transitioning the pellet from equilibrium at 4 % ± 2.5 RH to 95 % ± 2.5 RH, shows a continuous increase in capacitance over 6 min without reaching a limit. Conversely, desorption is rapid (25 s) with capacitance values returning to their original value (172 pF) when transitioning from 95 % ± 2.5 RH back to 4 % ± 2.5 RH. After 20 min of adsorption at 95 % ± 2.5 RH, the capacitance value reaches 310 pF and continues to rise while desorption remains swift taking less than 30 s to return to the initial value. Repetition of the experiment demonstrates the reproducibility of this behavior.

## 4 Conclusions

The humidity sensor based on MgTiO_3_ doped with 2 wt% of LiF was densified by Spark Plasma sintering process. The microstructure shows a low open porosity percentage (2 % ± 1) which allows to evaluate the sensitivity of this dense ceramic without the intervention of the phenomenon that can often occur in the pores in the case of conventional sintering [37].

However, the proportions of the phases formed at the surfaces of SPS pellets are probably different than the surfaces of the counterpart prepared by conventional sintering: MgTiO_3_ Rhombohedral phase, Face-Centered Cubic phase of LiF and Li_2_MgTiO_4_. We observed large grains on the surface attributed to the CFC Li_2_MgTiO_4_ phase generated on the surface of the pellets during the calcination step where the heat treatment time was enough to activate the coalescence of grains on the surface of the pellet.

The maximum densifications’ sintering temperature was lowered of 250 °C by the SPS as shown by the shrinkage versus temperature behavior of the ML2 where sintering temperature and time were lowered (800 °C sintering temperature and 30 min total sintering time) compared to conventional sintering (1050 °C sintering temperature and 15 h total sintering time) as investigated by dilatometric analysis.

ML2 pellets prepared by SPS at 800 °C are highly sensitive to moisture and it can be seen that the resistance of the sensor using ML2 SPS pellet changes by about three orders of magnitude compared to the sensor using conventional ML2 pellet at the same condition. The adsorption in a saturated atmosphere showed that the capacitance signal does not reach a limit at 95 % RH, however, desorption process is complete and quick (less than 30 s).

This improvement in the sensitivity of the SPS sample compared to the conventional sample sintered at 900 °C was attributed to the improvement of the skeletal density (respectively 3.92 ± 0.01 g cm^−3^ and 3.86 ± 0.01 g cm^−3^) and the lowering of the open porosity (respectively 2 % ± 1 and 17 % ± 1).

## Data availability statement

1. Has data associated with your study been deposited into a publicly available repository? NO
2. Why? Data will be made available upon request.

## CRediT authorship contribution statement

**Ahmad Kassas:** Writing – review & editing, Writing – original draft, Methodology, Investigation, Formal analysis. **Israa Zahwa:** Formal analysis. **Bassam Hussein:** Writing – review & editing, Resources. **Jérôme Bernard:** Validation, Supervision, Conceptualization. **Céline Lelièvre:** Supervision. **Mohamed Mouyane:** Methodology, Formal analysis. **Jacques Noudem:** Validation, Supervision, Formal analysis. **David Houivet:** Validation, Supervision, Resources, Conceptualization.

## Declaration of competing interest

The authors declare the following financial interests/personal relationships which may be considered as potential competing interests:Ahmad KASSAS reports financial support was provided by University of Caen Lower-Normandy Faculty of Sciences and Techniques of Physical activities and Sports. If there are other authors, they declare that they have no known competing financial interests or personal relationships that could have appeared to influence the work reported in this paper.
